# OSPAR: A Corpus for Extraction of Organic Synthesis Procedures with Argument Roles

**DOI:** 10.1021/acs.jcim.3c01449

**Published:** 2023-10-20

**Authors:** Kojiro Machi, Seiji Akiyama, Yuuya Nagata, Masaharu Yoshioka

**Affiliations:** † Graduate School of Information Science and Technology, Hokkaido University, Kita 14, Nishi 9, Kita-ku, Sapporo, Hokkaido 060-0814, Japan; ‡ Institute for Chemical Reaction Design and Discovery (WPI-ICReDD), Hokkaido University, Kita 21, Nishi 10, Kita-ku, Sapporo, Hokkaido 001-0021, Japan; § Faculty of Information Science and Technology, Hokkaido University, Kita 14, Nishi 9, Kita-ku, Sapporo, Hokkaido 060-0814, Japan

## Abstract

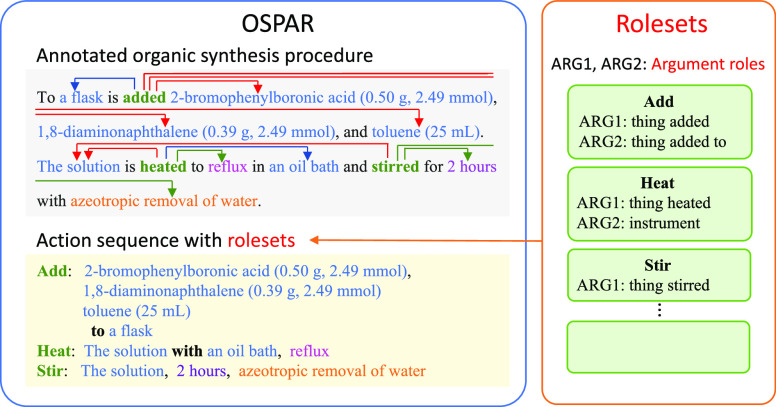

There is a pressing need for the automated extraction of chemical reaction information because of the rapid growth of scientific documents. The previously reported works in the literature for the procedure extraction either (a) did not consider the semantic relations between the action and argument or (b) defined a detailed schema for the extraction. The former method was insufficient for reproducing the reaction, while the latter methods were too specific to their own schema and did not consider the general semantic relation between the verb and argument. In addition, they did not provide an annotated text that aligned with the structured procedure. Along these lines, in this work, we propose a corpus named organic synthesis procedures with argument roles (OSPAR) that is annotated with rolesets to consider the semantic relation between the verb and argument. We also provide rolesets for chemical reactions, especially for organic synthesis, which represent the argument roles of actions in the corpus. More specifically, we annotated 112 organic synthesis procedures in journal articles from *Organic Syntheses* and defined 19 new rolesets in addition to 29 rolesets from an existing language resource (Proposition Bank). After that, we constructed a simple deep learning system trained on OSPAR and discussed the usefulness of the corpus by comparing it with chemical description language (XDL) generated by a natural language processing tool, namely, SynthReader. While our system’s output required more detailed parsing, it covered comparable information against XDL. Moreover, we confirmed that the validation of the output action sequence was easy as it was aligned with the original text.

## Introduction

The number of chemical reactions reported in scientific documents has rapidly increased. New chemical reactions have been collected in chemical reaction databases such as Reaxys^1^ and Scifinder^n^;^2^ however, this has been done by human experts and taken much time as well as high costs. To reduce this burden, the development of automated information extraction methods has been studied.^3−12^ While most of them are focused on core information, such as chemical entities and parameters, a few works have attempted to extract actions in a reaction from documents.

For example, the Cheminformatics Elsevier Melbourne Universities (ChEMU) campaign aimed to extract such chemical reaction data from patent documents.^9^ Because they are interested in the reaction information at the level of the reaction database, they did not consider detailed action information. For example, their corpus did not distinguish the semantic roles of A and B in the following phrase: “*A is added to B*” because they just needed a chemical reaction formula derived as A + B. However, the order of addition, namely, A to B or B to A, is usually regarded as important information that is required to reliably reproduce the chemical reaction.

There are two works in the literature for extracting more detailed procedures in organic chemistry. Mehr et al.^13^ developed a rule-based system called SynthReader to convert an experimental procedure to a chemical description language (XDL) that was compatible with their robotic platform, and they also proposed a platform for editing XDL. Their whole framework was an important step for the extraction of the experimental procedure. However, it is difficult to maintain a set of rules used in SynthReader against a wide variety of sentences. Vaucher et al.^14^ proposed a text-generation task that generated an action sequence from an experimental procedure. While their machine learning-based system trained on a large pseudocorpus and a small human-annotated corpus was more flexible and robust than rule-based systems, the users of their system had to read an original text to validate the generated procedure from various perspectives, such as safety and completeness. Therefore, we emphasize that not only are the structured data important but also the annotated text, which is aligned with the structured data.

**Figure 1:**
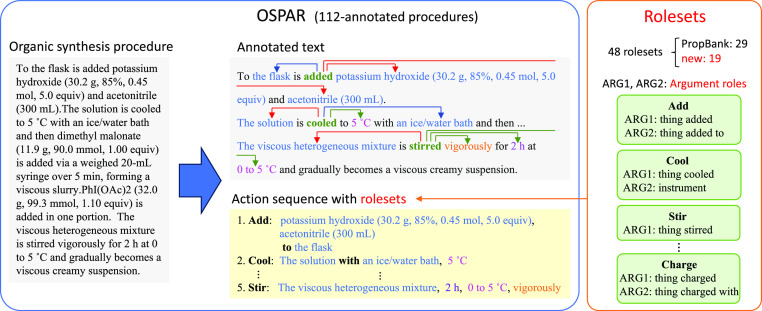
Overview of OSPAR and rolesets (verbs with argument roles). We annotated actions and related entities in the organic synthesis procedure as well as the semantic roles of the entities using rolesets. The rolesets were selected from PropBank if there were appropriate roles for the examples in the corpus. Otherwise, we defined the new rolesets. The procedure text is from ref (16).

To address these issues, we propose a corpus for procedure extraction named organic synthesis procedures with argument roles (OSPAR) that has more detailed information with annotated text than ChEMU’s corpus. ChEMU used semantic role labels based on Proposition Bank (PropBank)^15^ that represented relations between verbs and related terms. PropBank is a corpus to represent a set of semantic roles, which is constructed using *The Wall Street Journal*. Although ChEMU simplified the semantic labels, the PropBank and the simplified labels were not sufficient enough to describe the detailed procedure. Under this perspective, in this work, we systematically review the semantic role labels of the original PropBank and propose new semantic rolesets that are suitable for representing chemical reactions and especially organic synthesis. Our annotation schema using the augmented semantic role labels is more general than the one used in the specific platform (e.g., XDL), but it is easy to convert the information to the specific format. The overview of the roles of OSPAR and rolesets is shown in Figure 1. Because the texts in our corpus are aligned to the output of the action sequence, the users can validate the action sequence easier than the text-generation approaches.

In addition, we train a deep learning-based system using the corpus to evaluate the practical use of the corpus and conduct more detailed analyses. To elaborate on the generality and application possibility of our framework, we compared the output sequence of our corpus with an XDL example.

## Expansion of PropBank and Corpus Construction

### Semantic Roles and PropBank

Semantic roles express the relation between a predicate (verb) and its arguments. PropBank^15^ is one of the data sets annotated with semantic roles by using *The Wall Street Journal*. PropBank defines the semantic roles of verb senses individually and assigns a set of roles with numbers (Arg0, Arg1, Arg2, ...) to each verb sense (this is called roleset). ARG0 generally represents the agent of the predicate, and Arg1 represents the patient. Other arguments more than Arg2 do not have consistent usage.

This is an example of roleset.

- add.02
  1 ARG0: adder.
  2 ARG1: thing being added
  3 ARG2: thing being added to

If we apply this roleset to the sentence “*A is added to B*”, A is ARG1 and B is ARG2. Because one verb can have multiple rolesets, the number after the verb shows the index number of the roleset. While ARG0 shows the agent, we basically ignored this label in this work because the agent of action is obviously the experimenter. If rolesets are appropriately defined, the roles could be mapped to any schema for representing chemical actions, similar to the arguments for functions in computer programming.

Because *The Wall Street Journal* articles take news related to business and economics, the rolesets in PropBank cannot cover actions in chemistry. Therefore, we need to add new rolesets for chemistry concurrently with the construction of our corpus.

### Task Definition

**Figure 2:**
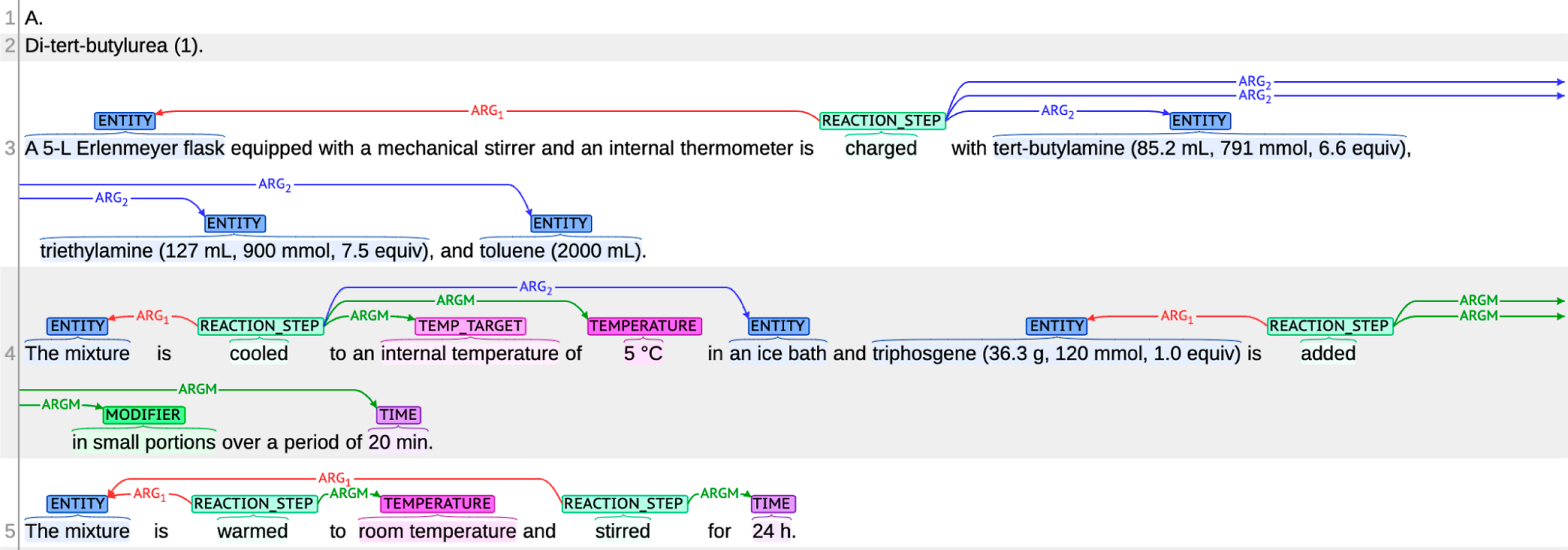
Example of annotation visualized by brat annotation tool^17^ (the procedure text is from ref (18)). The brat is used for visualization of annotation after this.

In this work, we focus on chemical reaction procedures in organic synthesis at the level of rolesets. While the procedures usually consist of synthesis and workup parts, we only used the synthesis part of the procedures. There were two reasons for this. (1) We focus on whether the product can be reproduced from the described procedure. Therefore, the method of workup, which affects the yield of the products, is not important. (2) Annotating workup parts requires considerably more cost than the synthesis parts because the vocabulary in the workup is much wider than that in synthesis. The preparation methods of instruments before starting a reaction (e.g., drying the instruments) are also out of the scope of this work. This is because experimenters do not necessarily follow the methods, as long as the state of the instruments is satisfied. Figure 2 shows an example of annotation. Our corpus consists of two tasks: named entity recognition (NER) for finding spans of entities, actions, and parameters, and relation extraction (RE) for finding relations between the action and entity/parameter.

### Rolesets for Actions in Chemistry

As we discussed, rolesets defined in PropBank are not sufficient for describing the action in chemistry. Therefore, we propose new rolesets for actions in chemistry based on PropBank. We checked the existence of appropriate entries in the PropBank for all actions in the corpus. If we could find appropriate entries, then these entries were included in our rolesets. However, there are several cases in the textual explanation that are about arguments that were not appropriate for describing chemical reactions. In such cases, we modified the text to reduce the ambiguity and to interpret the meaning of the argument. While the rolesets in PropBank sometimes contained more than three n of ARGn, the rolesets in our corpus had up to two, which was enough to represent examples in the corpus. We ordered them to smaller numbers because labels with fewer examples harm the performance of machine learning-based systems. As a result, the rolesets in our corpus contained only ARG1 and ARG2 as ARGn. When there were no appropriate rolesets, we defined new rolesets.

### Annotation Schema

We used six labels for NER and four labels for RE. While there were four NER labels (REACTION_STEP, ENTITY, TIME, TEMPERATURE), which had the same name as labels in ChEMU tasks,^9,19^ the definitions and target entities of the four labels were different from ChEMU’s labels.

Annotation labels for the NER are the following:

- REACTION_STEP: actions to transform the reactant to a product, except for the actions for the preparation of instruments before starting a reaction and workup. When there were parallel actions, such as “**added** with **stirring**”, **added** was annotated as REACTION_STEP, but **stirring** was annotated as MODIFIER because the relation between actions is out of scope in this corpus for simplification.
- ENTITY: entities that have a semantic relation with a REACTION_STEP. The entities include chemical substances, gas, flasks, and other instruments.
- TIME: time that is related to REACTION_STEP. This contains the exact time and duration, such as **14–15h**. Expression in words, such as **overnight,** is also annotated.
- TEMPERATURE: the temperature that is related to REACTION_STEP. This contains not only the exact temperature but also the range, such as **between 0** °**C and 5** °**C**. Expression in words, such as **room temperature,** is also annotated. In addition, if a specific temperature is not described but there is a word that indirectly represents temperature, such as **ice/water**, the word is annotated with this label.
- TEMP_TARGET: place to maintain TEMPERATURE. For example, **internal temperature** (inside the reactor) and **bath temperature**.
- MODIFIER: other information that is important to perform actions, including atmosphere, way to add compounds, stirring rate, parallel actions, and others. For example, under **nitrogen**, added **in one portion**, added **dropwise**.

In addition to each label’s definition, there are two policies for the annotation. The first one is if the same parameter was mentioned multiple times; only the first appeared parameter was annotated because the repetition was confusing in the conversion to the action sequence. For example, the second “**12h**” in “...was stirred **12h**. After **12h**, ...” was not annotated. The second is that the same entities and parameters, such as coreference and the amount of entities, are annotated as one span. For example, “12-aminododecanolactam (**1**) (9.00 g, 45.6 mmol, 1.00 equiv)”, (**1** is coreference of 12-aminododecanolactam) and “room temperature (23 °C)”.

Annotation labels for RE are the following:

- ARG1, ARG2: semantic relation between REACTION_STEP and ENTITY. The semantic relation depends on each roleset, but these labels are shared among the rolesets.
- ARGM: semantic relation between REACTION_STEP and parameters (TIME, TEMPERATURE, TEMP_TARGET, MODIFIER). We annotated the relation as ARGM even if the parameters were defined as ARGn in PropBank.
- (ARG0): semantic relation between REACTION_STEP and ENTITY. This represents the agent of an action. We basically did not use this label because most of the actions’ agents are experimenters. However, we used only one case, ENTITY A of “A **contain** B”, because ARG0 should be required to represent the container of the action.

### Data Curation and Preprocessing

Organic syntheses reported in scientific journals should be reproducible from the description of the experimental procedure. If the description does not provide enough information to perform the reaction, then experimenters cannot obtain the same result. *Organic Syntheses*(20) is one of the most reliable journals in organic synthesis because each procedure is checked carefully for reproducibility in the laboratory of a member of the Board of Editors. The editors or their colleagues carefully checked the reproducibility of each reaction by performing the reaction in their laboratory. Therefore, we decided to construct our corpus using the procedures in this journal.

We collected articles that had a “Procedure” section that described organic synthesis procedures, from annual volumes 81 (2005) to 97 (2020). While some articles contain multiple reactions to obtain the final product, we used only the procedure of the first reaction of each article for the corpus. This is because coreference resolutions were required if we handled procedures after the first reaction. In addition, we excluded procedures that have multiple boundaries of synthesis and workup. Finally, we obtained 112 procedures (Table S1). See the Supporting Information for article selection details.

We applied one preprocessing for the texts that were distinctive in *Organic Syntheses*. Here is an example phrase: *“anhydrous CH*_2_*Cl*_2_*(150 mL) (Note 11) (Figure 1)”*. As shown in this example, additional information, such as *Notes* and *Figures*, was written down in the raw texts. This is not common in other journals; thus, we removed these strings by using regular expressions.

### Roleset Expansion and Corpus Annotation

We constructed the OSPAR and developed rolesets against actions in the corpus. The corpus was annotated by using the brat annotation tool.^17^ This was done by three authors, two organic chemists (associate and assistant professor), and one information scientist (Ph.D. student).

First, we randomly selected 30 procedures to stabilize the annotation guideline. To reduce annotation costs, we used our system for ChEMU 2022 tasks as preannotation because several entities of our corpus had similar characteristics to ChEMU tasks. Then, we repeatedly annotated them and revised the guidelines. Next, we trained our system mentioned in the below section by using 30 procedures and deployed the system for the remaining 82 procedures as preannotation. Finally, we annotated the remaining procedures with the stabilized guideline.

The rolesets were added or modified for REACTION_STEP verbs concurrently with the annotation process. The modification included simple textual modification and the edit of the roles, which were affected by the change of the definition of arguments in our corpus from PropBank. For example, “ingredient one” (ARG1) and “ingredient two” (ARG2) from “mix.01” in PropBank were aggregated as “ingredient” (ARG1) in our corpus because the difference of ingredients in PropBank came from a syntactic perspective, but it was not important in terms of chemistry. When we searched rolesets in PropBank, we used the natural language toolkit (NLTK).^21^ To distinguish verbs regardless of the tense, we used Wordnet lemmatizer^22^ via NLTK. However, several REACTION_STEP could not be lemmatized when the verbs were not registered in Wordnet lemmatizer or written in noun form, such as **addition**. Therefore, we constructed a dictionary for these words. Finally, all REACTION_STEP in the corpus was given any roleset. While verbs in PropBank can have multiple rolesets, there was no verb that has multiple rolesets in the corpus. All rolesets used in the corpus were given independent suffix numbers from PropBank and numbered as 01.

To separate the synthesis and the workup parts, we defined the boundary as the end of the chemical transformation from the reactant to the product. In general, operations after the chemical transformation can be regarded as workup operations.

### Corpus Statistics

The annotated procedures were split into a training set, a development set, and a test set at a ratio of 8:1:1. Table 1 shows the number of procedures and sentences in each data set. Here, the number of sentences included only the synthesis part of a procedure.

**Table 1:** Number of Procedures and Sentences in Each Data Set

data set	procedure	sentence
train	90	664
dev.^a^	11	86
test	11	68
total	112	818

a Dev.: development.

Table 2 shows the number of NER labels in each data set. While REACTION_STEP and ENTITY had many examples, the parameters had fewer examples, especially TEMP_TARGET.

**Table 2:** Number of NER Labels in Each Data Set

label	train	dev.	test
REACTION_STEP	590	66	59
ENTITY	900	108	100
TEMPERATURE	225	27	21
TEMP_TARGET	48	10	4
TIME	218	31	24
MODIFIER	161	17	15
total	2142	259	223

Table 3 shows the number of RE labels in each data set. ARG2 had fewer examples than the others because some rolesets did not have ARG2, and ARG2 sometimes was not used by REACTION_STEP even if ARG2 was included in the roleset.

**Table 3:** Number of RE Labels in Each Data Set

label	train	dev.	test
ARG1	611	68	61
ARG2	361	48	44
ARGM	648	81	63
ARG0^a^	1	0	0
total	1621	197	168

a ARG0 was only used for **contain.01**.

### Roleset Analysis

We obtained 48 rolesets from the corpus (Table S2). To evaluate the effectiveness of roleset expansion, we classified them into four types:

A Same as original PropBank or slightly modified string surface, or reduced and ordered arguments of the existing roleset in PropBank.
B Affected by the change in the definition of arguments from PropBank.
C New roleset but the verb existed in PropBank.
D New roleset and the verb did not exist in PropBank.

Table 4 shows the distribution of types for the top 15 frequent rolesets and all rolesets. There were 52% of (A) rolesets and 40% of newly defined (C + D) rolesets. Therefore, the vocabulary of PropBank was insufficient for chemistry, and we confirmed the importance of expanding rolesets for chemistry.

**Table 4:** Distribution of the Types of Rolesets

	(A)	(B)	(C)	(D)
top 15	7	0	5	3
all (48)	25	4	11	8

**Figure 3:**
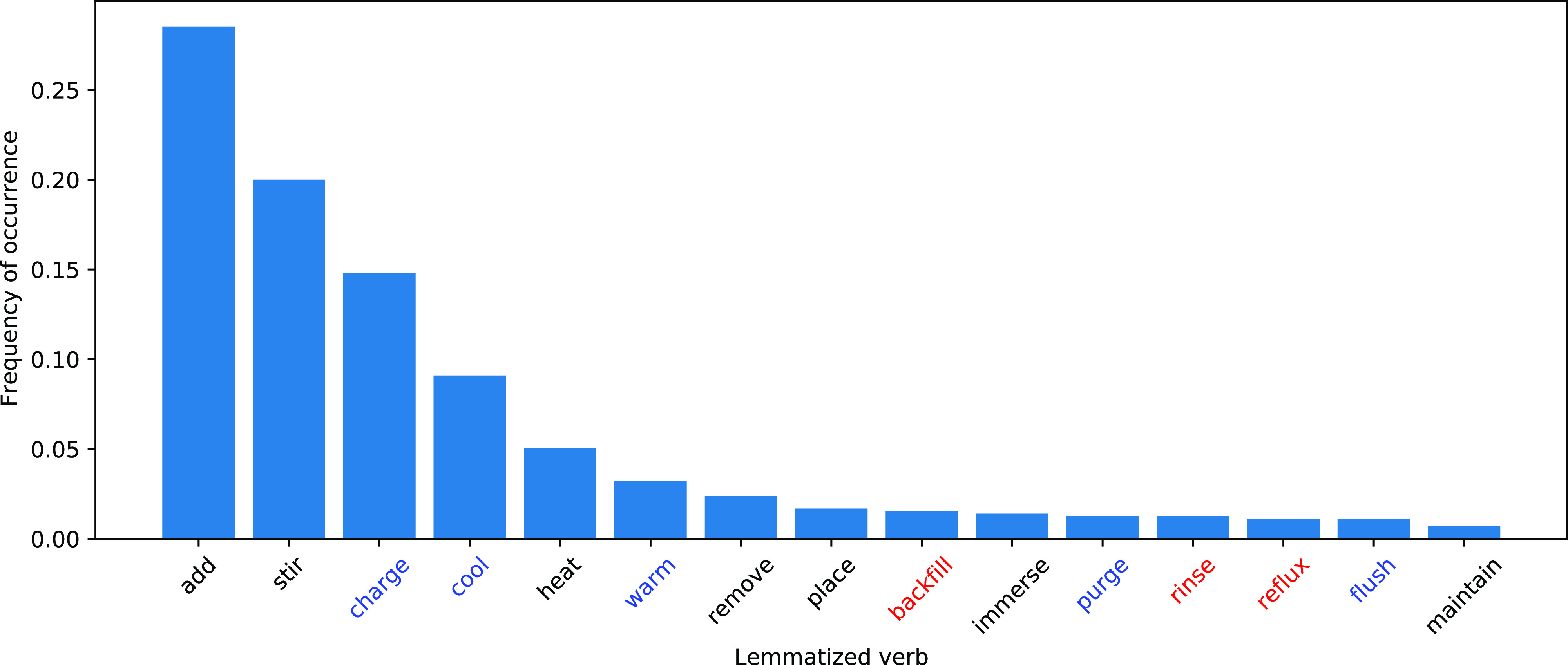
Distribution of rolesets in the corpus (most frequent 15). The colors of verbs show the types of roleset: (A) black, (C) blue, and (D) red. [There was no (B)].

Figure 3 shows the distribution of rolesets in the corpus. We confirmed that the distribution of verbs was unbalanced, as shown in related works. This is because the actions in organic synthesis are limited. It is worth noting that **charge** and **cool**, which were the third and fourth frequent rolesets, were categorized into (C).

## Experiment

We constructed an information extraction system by using the OSPAR to verify the practical use of the corpus and conduct a more detailed analysis.

### System Description

Recent deep learning models with pretraining have shown remarkable performances in natural language processing tasks.^23−25^ We developed a pipeline system for NER and RE tasks that used ChemBERT v3.0,^11^ a pretrained language model for chemistry, similar to our previous work for the ChEMU 2022 task.^26^

**Figure 4:**
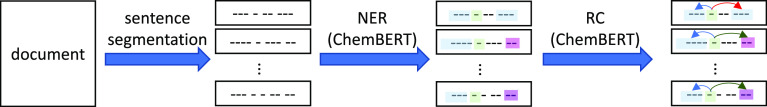
Overview of the pipeline system.

Figure 4 shows the pipeline method. First, we split a document into sentences by using ChemDataExtractor.^7^ Then, we used ChemBERT models for NER and another ChemBERT model for RE. We used AllenNLP^27^ and Hugging Face Transformers^28^ for implementing ChemBERT.

For NER, we trained three ChemBERT models for: ENTITY, REACTION_STEP, and parameters. This is because each label can be overlapped, and a simple ChemBERT model cannot take the overlapped entities. The input was tokens in a sentence that was split by simple regex rules.^29^ The output was a sequence of IOB2 labels with a class of each token. The labels were predicted in the same method as the original BERT.^24^ For the optimization in training, AdamW optimizer^30^ and cross-entropy loss were used. The hyperparameter values for the training are the same as our previous work:^26^ max sequence length = 384 (this value is enough to cover all sequences contained in the training and development sets), batch size = 16, learning rate = 1 × 10^–5^. We applied early stopping at the training with patience = 7 and used the best *F*-score model on the development set for evaluation.

For RE, we trained one ChemBERT model. The model solved the RE task as a classification problem of the relation of two entities.^31^ ChemBERT predicted the labels of all candidate pairs in a sentence. The input of ChemBERT was a sentence with special tokens; a REACTION_STEP was [E1] and [/E1] tokens, and an ENTITY or a parameter was enclosed by [E2] and [/E2] tokens. The output was a relation label or the label to show that there was no relation. In the training stage, we used gold-standard entities of the corpus. Other settings were the same as NER.

### Evaluation Metrics

We used precision, recall, and *F*-score for evaluation of the system.
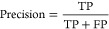

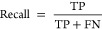

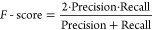
where TP is true positives, which are correctly predicted by the system; FP is false positives, which are incorrectly predicted by the system; and FN is false negatives, which are not found by the system but exist in the corpus.

To evaluate close failures, we used a relaxed match for the evaluation of NER in addition to an exact match. The relaxed match means that the span of the entity is partially matched to the TP, and the label is correct.

## Results and Discussion

Table 5 shows the NER results on the test set.

**Table 5:** NER Results on the Test Set

	exact match			relaxed match			
label	precision	recall	*F*-score	precision	recall	*F*-score	examples
REACTION_STEP	0.9474	0.9153	0.9310	0.9474	0.9153	0.9310	59
ENTITY	0.8447	0.8700	0.8571	0.8932	0.9200	0.9064	100
TEMPERATURE	0.6786	0.9048	0.7755	0.7500	1.0000	0.8571	21
TEMP_TARGET	0.5000	0.7500	0.6000	0.6667	1.0000	0.8000	4
TIME	0.9231	1.0000	0.9600	0.9231	1.0000	0.9600	24
MODIFIER	0.8571	0.8000	0.8276	0.9286	0.8667	0.8966	15
all	0.8504	0.8924	0.8709	0.8889	0.9327	0.9103	223

While our corpus was not large, the NER model obtained an *F*-score of 0.8709 in an exact match. The model obtained an *F*-score of 0.9103 in a relaxed match, which was 0.04 times greater than the exact match. This means the existence of some errors in the detection of the entities’ spans.

**Figure 5:**
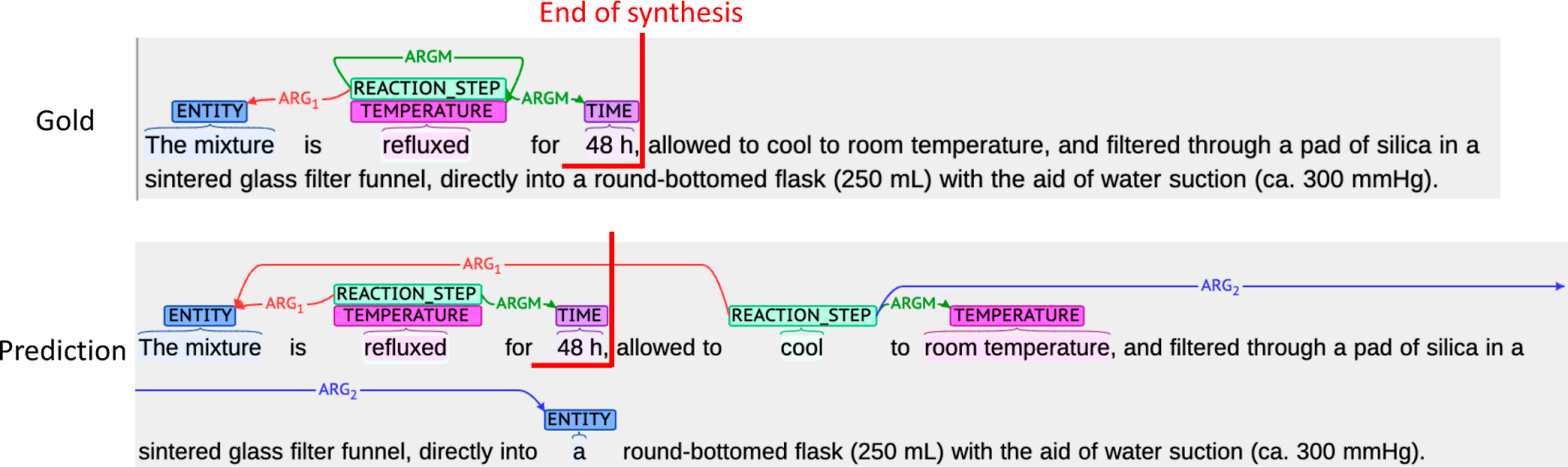
Incorrect prediction due to the boundary of synthesis and workup.

The precision was relatively lower than that of the recall. One of the reasons was that there were a few sentences that contained both synthesis and workup actions. In such cases, the model incorrectly predicts workup actions and related entities, even though the actions for the workup are not annotated in the corpus (Figure 5).

Table 6 shows the RE results on the test set.

**Table 6:** Relation Classification Results on the Test Set

entity	precision	recall	F-score
predicted	0.8393	0.8393	0.8393
gold standard	0.9408	0.9464	0.9436

The model with predicted entities obtained an *F*-score of 0.8393 in an exact match. On the other hand, the model with gold-standard entities obtained an *F*-score of 0.9436 in an exact match. We recognized the importance of NER performance, and this difference (approximately 0.1) was not small. On the other hand, the results of NER and RE suggest the consistency of the annotation of the corpus and the usefulness of the corpus.

As expected, our system tends to fail in extracting entities that are seen zero or a few times during training. On the other hand, we found interesting cases in that our system succeeded in finding such entities. Table 7 shows the recalls of each verb (REACTION_STEP) on the test set.

**Table 7:** Recalls of Each Verb (REACTION_STEP) on the Test Set^a^

verb	recall	#test	#train	#dev.
add	1.0	18	168	18
stir	1.0	12	119	12
charge	1.0	8	86	12
cool	1.0	4	57	4
heat	1.0	3	31	2
remove	1.0	1	16	0
backfill	1.0	1	9	1
place	1.0	2	9	1
reflux	1.0	1	4	3
transfer	0.0	1	3	0
dissolve	0.0	1	3	0
fill	1.0	1	1	0
wrap	1.0	1	1	0
wash	0.0	1	1	0
**hold**	**1.0**	**1**	**0**	**0**
**mix**	**1.0**	**1**	**0**	**0**
open	0.0	1	0	0
activate	0.0	1	0	0

a #test, #train, and #dev. are the number of occurrence in each data set.

**Figure 6:**
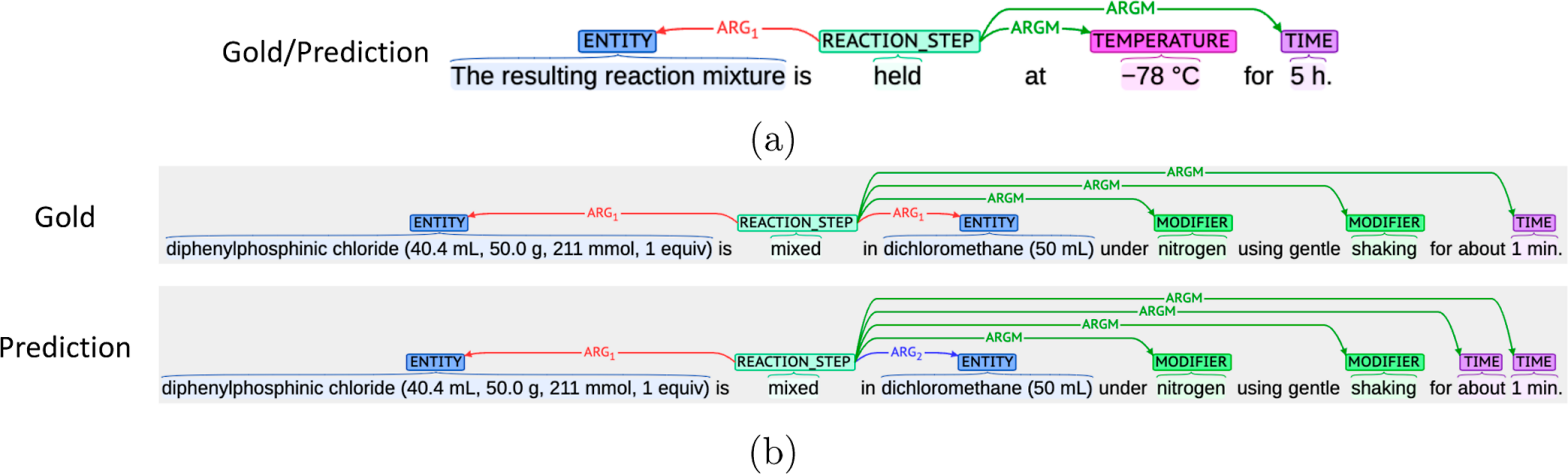
Prediction results for the unseen verbs (a) hold and (b) mix.

All verbs that existed more than three times on the train set were found in the test set. In fewer verbs, “fill” and “wrap” were found, even though the system saw them only once in the training phase. Although “hold” and “mix” were not seen in the training phase, they were found by the system. Figure 6 shows the prediction results for the unseen verbs. “Hold” was exactly matched in both NER and RE. This could be explained because similar cases were included in the train set, for example, “keep” and “maintain”. Although the roleset “mix.01” consists of only ARG1 (“thing mixed”), ARG2 was predicted for “dichloromethane (50 mL)” (we did not distinguish ingredients one and two in the roleset “mix.01” because the differences were not important in the context of conducting the synthesis procedure). This may be caused by similar examples, such as “A was heated in B” and “A was cooled in B”. In these cases, A is ARG1 (thing heated/thing cooled), and B is ARG2 (instrument). Regardless of the predicted labels, the prediction can be corrected by referring to the arguments of the roleset.

### Comparison with XDL

**Figure 7:**
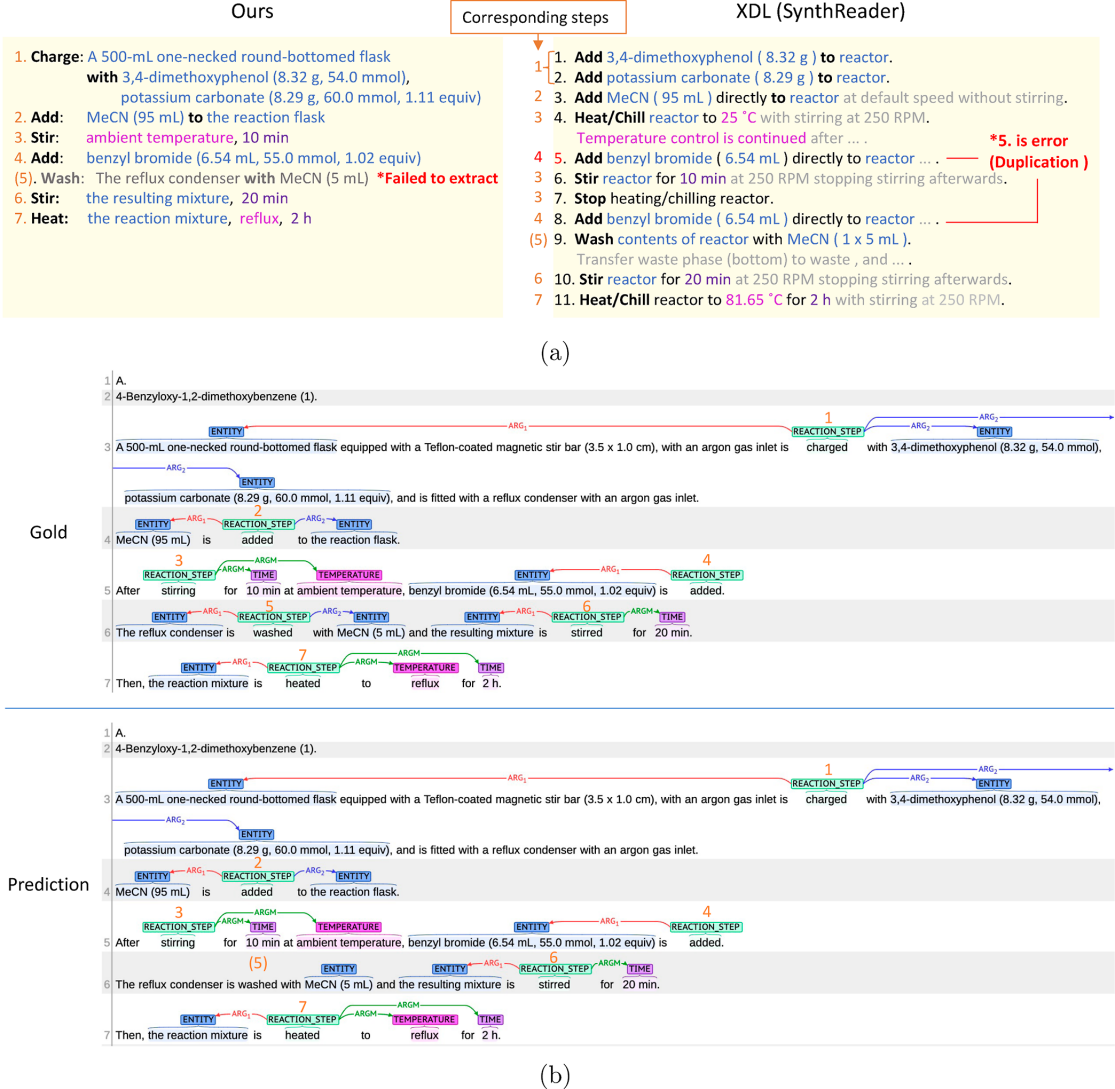
Comparison between our method and XDL generated by SynthReader (the procedure text is from ref (18)). (a) Action sequence for both methods. Here, some detailed explanation for robotic synthesis in XDL was omitted for simplicity of comparison. (b) Gold standard and prediction by our system visualized by using brat. The numbers over REACTION_STEP were manually drawn by us.

To investigate the completeness of our schema, we selected one example from the extracted sequence by our system and compared it with XDL generated by using SynthReader via ChemIDE.^32^ SynthReader is an information extraction system for XDL and showed high performance for articles in *Organic Syntheses*.^20^Figure 7 shows a comparison between our method and XDL generated by SynthReader.

We aligned the corresponding steps from ours and XDL (Figure 7a). Here, our system failed to extract the fifth action “Wash”, and SynthReader wrongly generated duplicated actions (the fifth and eighth action “Add” the fifth one was incorrect). By assuming that both systems could correct the action sequence, ours can be basically mapped to the actions in the XDL sequence. This implies that our schema with argument roles has the potential to represent general and sufficient information for organic synthesis.

It is important to align the annotation with the text. Figure 7b shows the visualized texts of the gold standard and prediction. If we verify the predicted action sequence, we can easily understand actions, related entities, and the failure of extraction. XDL by SynthReader contained more detailed information than ours; for example, the temperature of the final action “reflux” was interpreted as “81.65 °C” by using chemical knowledge. However, there are some mistakes in the XDL. As mentioned above, the fifth action, “Add”, was not required. In addition, although the ninth action, “Wash”, should be rinsing for the reflux condenser, it was interpreted as a separation action for compounds in the reactor. That is usually performed as workup.

### Limitations and Future Works

We have discussed the rolesets in the context of organic synthesis so far. We consider that it can be used in materials science^33−38^ because there are common actions in organic synthesis and materials science. On the other hand, we found that the variety of the rolesets is insufficient to represent actions in materials science. In such a case, we should expand rolesets for them. To enable this, we hope that not only our group but also other research groups will use this framework and enrich the rolesets and annotated corpus.

Finally, we discuss the limitations of this work. Because the relations among ENTITY in the corpus were unclear, anaphora resolution^39^ (the problem of resolving what a pronoun or a noun phrase refers to) should be required to generate structured data for performing the reaction. Most such relations are relations between a chemical compound and a mixture. One of the solutions is applying the task that is similar to ChEMU’s anaphora resolution task.^19^ Another challenge is interpreting ENTITY for structured data. For example, distinguishing instruments and chemical compounds. In addition, the amount of chemical compounds should be parsed. To enable this, ChemicalTagger,^40^ a rule-based natural language processing parser for chemistry, can be used. Although ChemicalTagger is less flexible than deep learning-based systems, it can extract detailed information, such as amount and instruments, which follows notions included in its rules. Therefore, it can be useful for interpreting information in ENTITY. Next, the variation and amount of examples in the corpus could be insufficient. We need to study the actions in different documents and compare them with the existing rolesets. In addition, we should increase the number of examples of rare rolesets.

## Conclusions

To understand the relation between actions in a chemical reaction procedure and the argument roles, we propose the rolesets in chemistry that can be used as a general procedure extraction task in organic/inorganic chemistry. We constructed the corpus named the OSPAR and evaluated it by training the system on the corpus. We confirmed the roleset was promising for expressing actions in reproducing a reaction by comparing it with XDL. On the other hand, we have found the vocabulary of the rolesets is still limited, and it should be extended in the future. While there were several errors due to the limited amount of the corpus, they can be resolved by increasing the number of corpora with the roleset.

In the future, we will check whether our schema can be used as a basic step in specific tasks by making a translation system from our schema to the program of robotic synthesis.^41,42^ In addition, the rolesets must be extended, and the system for the semantic relation extraction task should be developed. We hope the roleset will be maintained by the community, and the community will share their own corpus with the semantic relation by using the roleset.

## Supporting Information Available

Details of the article selection for OSPAR and the list of the rolesets (PDF)

## Notes

The OSPAR data set and the rolesets are available on GitHub.^43^
