# Supplementary material for: OSPAR: A Corpus for Extraction of Organic Synthesis Procedures with Argument Roles
Source: J Chem Inf Model. 2023 Oct 20;63(21):6619–28. doi: 10.1021/acs.jcim.3c01449 (PMC10647022; doi:10.1021/acs.jcim.3c01449)
Supplement: Supplementary file 1 — ci3c01449_si_001.pdf [file ci3c01449_si_001.pdf]

Supporting Informaton for OSPAR: A Corpus for Extraction of Organic Synthesis Procedure with Argument Roles

Kojiro Machi,[*,†] Seiji Akiyama,[‡] Yuuya Nagata,[‡] and Masaharu Yoshioka[*,†,¶,‡]

†*Graduate School of Information Science and Technology, Hokkaido University, Kita 14 Nishi 9, Kita-ku, Sapporo, Hokkaido, 060-0814, Japan*

‡*Institute for Chemical Reaction Design and Discovery (WPI-ICReDD), Hokkaido University, Kita 21 Nishi 10, Kita-ku, Sapporo, Hokkaido 001-0021, Japan*

¶*Faculty of Information Science and Technology, Hokkaido University, Kita 14 Nishi 9, Kita-ku, Sapporo, Hokkaido, 060-0814, Japan*

E-mail: machi@eis.hokudai.ac.jp; yoshioka@ist.hokudai.ac.jp

Article selection

This section describes the details of article selection in Section "Data curation and preprocessing." First, we collected articles from annual volumes of *Organic Syntheses*[1] 81 (2005) to 97 (2020). Next, we selected articles that had a "Procedure" section, and we used only the procedure of the first reaction of each article for the corpus. In addition, we excluded procedures that have multiple boundaries of synthesis and work-up.

As we internally constructed a corpus for the extraction of chemical entities in the past, we used the same articles in this work. Most articles in *Organic Syntheses*[1] have the annotation of chemical names with CAS (Chemical Abstructs Service) number. In the previous work, we

limited the documents whose all chemical names were aligned with the entries in Reaxys[2] for the preannotation of the corpus. We used Chemical Identifier Resolver (CIR)[3] via CIRpy[4] to identify the CAS number of chemical names if there was no CAS number in the article of *Organic Syntheses*.

Finally, we obtained 112 procedures as shown in Table S1.

Rolesets

Table S2 shows the list of the rolesets used for our corpus. The column "Type" indicates the following types:

(A). Same as original PropBank or slightly modified string surface, or reduced and ordered arguments of the existing roleset in PropBank.

(B). Affected by the change in the definition of arguments from PropBank.

(C). New roleset but the verb existed in PropBank.

(D). New roleset and the verb did not exist in PropBank.

References

(1) *Organic Syntheses*. `http://www.orgsyn.org`, (accessed October 14, 2021).

(2) Reaxys. `https://www.reaxys.com`, (accessed June 20, 2023).

(3) CIR. `https://cactus.nci.nih.gov/chemical/structure`, (accessed October 14, 2021).

(4) CIRpy. `https://github.com/mcs07/CIRpy`, (accessed October 14, 2021).

Table S1: Paper list of the OSPAR

Org. Synth. **2005**, 81, 1	*Org. Synth.* **2009**, 86, 262	*Org. Synth.* **2016**, 93, 263
Org. Synth. **2005**, 81, 14	*Org. Synth.* **2009**, 86, 298	*Org. Synth.* **2016**, 93, 272
Org. Synth. **2005**, 81, 26	*Org. Synth.* **2009**, 86, 308	*Org. Synth.* **2016**, 93, 413
Org. Synth. **2005**, 81, 63	*Org. Synth.* **2009**, 86, 315	*Org. Synth.* **2017**, 94, 16
Org. Synth. **2005**, 81, 244	*Org. Synth.* **2009**, 86, 325	*Org. Synth.* **2018**, 95, 97
Org. Synth. **2005**, 82, 1	*Org. Synth.* **2010**, 87, 16	*Org. Synth.* **2018**, 95, 112
Org. Synth. **2005**, 82, 30	*Org. Synth.* **2010**, 87, 126	*Org. Synth.* **2018**, 95, 276
Org. Synth. **2005**, 82, 64	*Org. Synth.* **2010**, 87, 192	*Org. Synth.* **2018**, 95, 310
Org. Synth. **2005**, 82, 93	*Org. Synth.* **2010**, 87, 218	*Org. Synth.* **2018**, 95, 357
Org. Synth. **2005**, 82, 99	*Org. Synth.* **2010**, 87, 310	*Org. Synth.* **2018**, 95, 425
Org. Synth. **2005**, 82, 179	*Org. Synth.* **2011**, 88, 398	*Org. Synth.* **2019**, 96, 36
Org. Synth. **2006**, 83, 18	*Org. Synth.* **2013**, 90, 62	*Org. Synth.* **2019**, 96, 418
Org. Synth. **2006**, 83, 31	*Org. Synth.* **2013**, 90, 164	*Org. Synth.* **2019**, 96, 436
Org. Synth. **2006**, 83, 49	*Org. Synth.* **2013**, 90, 229	*Org. Synth.* **2019**, 96, 455
Org. Synth. **2006**, 83, 61	*Org. Synth.* **2013**, 90, 240	*Org. Synth.* **2020**, 97, 12
Org. Synth. **2006**, 83, 70	*Org. Synth.* **2013**, 90, 287	*Org. Synth.* **2005**, 81, 157
Org. Synth. **2006**, 83, 97	*Org. Synth.* **2013**, 90, 306	*Org. Synth.* **2005**, 82, 134
Org. Synth. **2006**, 83, 103	*Org. Synth.* **2013**, 90, 327	*Org. Synth.* **2008**, 85, 138
Org. Synth. **2006**, 83, 111	*Org. Synth.* **2013**, 90, 338	*Org. Synth.* **2008**, 85, 189
Org. Synth. **2006**, 83, 155	*Org. Synth.* **2014**, 91, 39	*Org. Synth.* **2009**, 86, 92
Org. Synth. **2006**, 83, 193	*Org. Synth.* **2014**, 91, 93	*Org. Synth.* **2009**, 86, 344
Org. Synth. **2007**, 84, 22	*Org. Synth.* **2014**, 91, 106	*Org. Synth.* **2012**, 89, 230
Org. Synth. **2007**, 84, 32	*Org. Synth.* **2014**, 91, 116	*Org. Synth.* **2016**, 93, 163
Org. Synth. **2007**, 84, 43	*Org. Synth.* **2015**, 92, 1	*Org. Synth.* **2016**, 93, 352
Org. Synth. **2007**, 84, 68	*Org. Synth.* **2015**, 92, 13	*Org. Synth.* **2018**, 95, 486
Org. Synth. **2007**, 84, 88	*Org. Synth.* **2015**, 92, 117	*Org. Synth.* **2020**, 97, 139
Org. Synth. **2007**, 84, 262	*Org. Synth.* **2015**, 92, 182	*Org. Synth.* **2005**, 82, 157
Org. Synth. **2007**, 84, 272	*Org. Synth.* **2015**, 92, 195	*Org. Synth.* **2005**, 82, 170
Org. Synth. **2007**, 84, 295	*Org. Synth.* **2015**, 92, 247	*Org. Synth.* **2010**, 87, 115
Org. Synth. **2007**, 84, 325	*Org. Synth.* **2015**, 92, 267	*Org. Synth.* **2014**, 91, 27
Org. Synth. **2007**, 84, 347	*Org. Synth.* **2015**, 92, 296	*Org. Synth.* **2014**, 91, 60
Org. Synth. **2007**, 84, 359	*Org. Synth.* **2015**, 92, 356	*Org. Synth.* **2015**, 92, 171
Org. Synth. **2008**, 85, 34	*Org. Synth.* **2016**, 93, 75	*Org. Synth.* **2015**, 92, 227
Org. Synth. **2008**, 85, 172	*Org. Synth.* **2016**, 93, 100	*Org. Synth.* **2015**, 92, 328
Org. Synth. **2009**, 86, 11	*Org. Synth.* **2016**, 93, 115	*Org. Synth.* **2016**, 93, 63
Org. Synth. **2009**, 86, 18	*Org. Synth.* **2016**, 93, 178	*Org. Synth.* **2018**, 95, 127
Org. Synth. **2009**, 86, 36	*Org. Synth.* **2016**, 93, 245	*Org. Synth.* **2020**, 97, 54
Org. Synth. **2009**, 86, 252		

Table S2: List of rolesets

Roleset	ARG1	ARG2	ARG0	Type	PropBank
activate	thing activated			A	activate.01
add	thing added	thing added to		A	add.02
contain	contents		container	A	contain.01
dissolve	thing dissolved	liquid		A	dissolve.01
distill	thing distilled			A	distill.01
fill	thing filled	thing filled with		A	fill.01
heat	thing heated	instrument		A	heat.01
hold	thing held			A	hold.11
immerse	thing immersed	thing immersed in		A	immerse.01
inject	thing injected	place injected into		A	inject.01
introduce	thing introduced	place introduced into		A	introduce.01
keep	thing kept			A	keep.01
maintain	thing maintained			A	maintain.01
melt	thing melted			A	melt.01
open	thing opened			A	open.01
place	thing put	where put		A	place.01
pour	thing poured	thing poured into		A	pour.01
protect	thing protected	thing protected from		A	protect.01
remove	thing removed	thing removed from		A	remove.01
replace	old thing	new thing		A	replace.01
stir	thing stirred			A	stir.01
transfer	thing transferred	thing transferred to		A	transfer.01
use	thing used			A	use.01
wash	thing washed	liquid		A	wash.01
wrap	thing wrapped	thing wrapped in		A	wrap.01
combine	thing combined			B	combine.01
increase	thing increased			B	increase.01
mix	thing mixed			B	mix.01
raise	thing raised			B	raise.01
change	thing changed	thing changed to		C	
charge	thing charged	thing charged with		C	
chill	thing chilled	instrument		C	
collect	thing collected	thing collected in		C	
cool	thing cooled	instrument		C	
dilute	thing diluted	thing diluted with		C	
flush	target	liquid or gas		C	
load	thing loaded	thing loaded with		C	
prepare	thing prepared	thing prepared in		C	
purge	thing purged	thing purged with		C	
warm	thing warmed	instrument		C	
backfill	thing backfilled	thing backfilled with		D	
degas	thing degassed			D	
pour off	thing poured off			D	
reflux	thing refluxed			D	
rinse	thing rinsed	thing rinsed with		D	
swirl	thing swirled			D	
re-cool	thing recooled	instrument		D	
re-charge	thing recharged	thing recharged with		D	
